# Multistate Outbreak of *Salmonella* Infantis, Newport, and Lille Infections Linked to Live Poultry from a Single Mail-Order Hatchery in Ohio — March–September, 2012

**Published:** 2013-03-22

**Authors:** Tony M. Forshey, Scott Nowicki, Marika Mohr, C. Stephen Roney, Thomas M. Gomez, Jennifer R. Mitchell, Thai-An Nguyen, Casey Barton Behravesh

**Affiliations:** Ohio Dept of Agriculture; Ohio Dept of Health; US Dept of Agriculture; CDC

In early 2012, three clusters of human *Salmonella* infections were identified through PulseNet, a national network of public health and food regulatory agency laboratories coordinated by CDC that subtypes disease-causing organisms. Initial investigations indicated many of the ill persons in these three clusters had contact with live poultry (e.g., chicks and ducklings) from a single mail-order hatchery; therefore, the three investigations were merged. During March 1–September 24, 2012, a total of 195 persons infected with the outbreak strains of *Salmonella* serotypes Infantis, Newport, and Lille were reported from 27 states.

Among persons infected, 64 (33%) of 194 were aged ≤10 years; the age of one infected person was unknown. Seventy-nine (79%) of 100 ill persons who were interviewed reported contact with live poultry in the week before illness. Among 39 ill persons who purchased live poultry from the mail-order hatchery and who provided a reason for their purchase, all reported purchasing live poultry for backyard flocks to produce eggs or meat, or to keep as pets. Birds were purchased from multiple feed stores or directly from hatcheries. The median period from acquiring poultry and illness onset was 19 days (range: 3–90 days). Forty-seven (87%) of 54 ill persons with available purchase information reported buying chicks or ducklings sourced from a single mail-order hatchery in Ohio.

The mail-order hatchery is a participant in the U.S. Department of Agriculture’s National Poultry Improvement Plan (USDA-NPIP). This program is intended to eliminate certain strains of *Salmonella* that cause illness in poultry-breeding flocks and hatcheries. However, the program does not certify that these poultry are free from other strains of *Salmonella* that might cause illness in humans. Recently, the same mail-order hatchery has been linked to other human *Salmonella* infections outbreaks (1,2).

This outbreak investigation identified the largest number of human illnesses ever linked to contact with live poultry during a single outbreak, and it underscores the ongoing risk for human salmonellosis linked to backyard flocks. Preventing live poultry–associated salmonellosis requires an integrated approach involving mail-order hatcheries, agricultural feed stores, and consumers. Mail-order hatcheries should comply with management and sanitation practices outlined by USDA-NPIP and avoid the shipment of day-old chicks through their hatchery from another hatchery (e.g., trans-shipping). Feed stores should use physical barriers (e.g., a wall or fence) between customers and poultry displays to prevent direct contact with poultry (3). Educational materials warning customers and advising them on how to reduce the risk for *Salmonella* infection from live poultry should be distributed with all live poultry purchases (4).
